# Optimal production and purification of n.c.a.^143^Pr as a promising palliative agent for the treatment of metastatic bone pain

**DOI:** 10.1038/s41598-024-64321-z

**Published:** 2024-06-12

**Authors:** Simindokht Shirvani-Arani, Hassan Ranjbar, Ali Bahrami-Samani

**Affiliations:** Nuclear Fuel Cycle Research School, Nuclear Science and Technology Research Institute (NSTRI), Tehran, Iran

**Keywords:** Pure β-emitter, Non-carrier-added^143^Pr, Tehran research reactor, Extraction chromatography, Experimental nuclear physics, Biophysical chemistry, Radiotherapy, Targeted therapies

## Abstract

This study proposes the beta-emitting radioisotope ^143^Pr as a promising candidate for palliative treatment of metastatic bone pain due to its desirable physical decay characteristics. An optimized process was developed for the production and purification of non-carrier-added ^143^Pr using a medium flux research reactor. Calculations were performed to determine the optimal irradiation time and cooling period for irradiating 1 mg of natural cerium oxide to indirectly produce ^143^Pr through the decay of ^143^Ce. Following irradiation and cooling, extraction chromatography was employed to efficiently isolate ^143^Pr from the irradiated target material. A column containing Ln-resin was used along with nitric acid as the mobile phase and an optional oxidation step with NaBrO_3_/ascorbic acid to separate ^143^Pr from impurities such as ^143^Ce and ^141^Ce. Radionuclidic purity of over 99.995% was achieved as confirmed through gamma spectroscopy, demonstrating effective separation of ^143^Pr. Additional quality control analyses established the chemical and radiochemical purity of the purified ^143^Pr nitrate product. With a half-life of 13.6 days and maximum beta energy of 0.937 MeV, ^143^Pr exhibits favorable properties for palliative bone pain therapy. This study therefore provides a viable method for producing high-purity ^143^Pr through the optimized irradiation and purification processes described. Further investigation is warranted to explore potential clinical applications of ^143^Pr for palliation of metastatic bone cancer pain.

## Introduction

The most important beta emitter-radionuclides applied in the preparation of pain palliatives for metastatic bone include samarium-153 (^153^Sm)^1^, strontium-89 (^89^Sr)^2^, phosphorus-32 (^32^P)^3^, and lutetium-177 (^177^Lu)^4^, holmium-166 (^166^Ho)^5^, and rhenium-186 (^186^Re)^6^. These radionuclides have different physical characteristics and, therefore, different levels of effectiveness in relieving the pain associated with metastatic bone (Table 1)^7^. Naturally, a longer half-life results in a longer duration of efficacy and pain relief, such as when ^153^Sm and ^177^Lu are combined with EDTMP. The relatively low β-energy and also relatively long half-life of ^177^Lu allow the deposition of an adequate tumor irradiation dose at a constant rate^8–10^. ^32^P and ^89^Sr have long half-lives, but due to their high deposited energies, cells absorb high levels of the extra dose, and the bone marrow ablation is probable. Meanwhile, radionuclides with long half-lives present waste disposal issues in hospitals where various therapeutic actions are undertaken.

**Table 1:** Half-life, ${E}_{\beta max}$, ${E}_{\beta ave}$, and ${E}_{\gamma }$ of the most important beta emitter-radionuclides applied in the preparation of pain palliatives for metastatic bone.

Radionuclide	${T}_\frac{1}{2}$ (d)	${E}_{\beta max}$(MeV)	${E}_{\beta ave}$(MeV)	${E}_{\gamma }$(keV)
^153^Sm	1.93	0.81	0.233	103 (28%)
^89^Sr	50.5	1.46	0.583	–
^32^P	14.28	1.7	0.7	–
^177^Lu	6.73	0.489	0.133	113 (6%), 208 (11%)
^166^Ho	1.12	1.84	0.67	81 (7%), 1400 (~ 1%)
^186^Re	3.7	1.07	0.349	137 (9%)
^143^Pr	13.57	0.934	0.315	–

The half-life of ^143^Pr is 2 weeks, which is longer than the half-life of ^177^Lu, ^153^Sm, and ^186^Re, while it is similar to that of ^32^P. It has pure beta emission with an E_βmax_ of 0.937 and an MeV lower than ^89^Sr and ^32^P too. Pure beta emitter ^33^P, also shows suitable properties such as E_βmax_ = 0.2 MeV and a half-life of 25 days; however its production is limited by needing high flux reactor for some countries. Thus, ^143^Pr is proposed here as a promising radionuclide for preparing bone metastases pain palliatives that allow for an adequate deposition of irradiation resulting in reasonable efficacy, along with a proper half-life and energy. In addition, ^143^Pr can be produced in a no-carrier-added form in a medium flux reactor, such as the Tehran Research Reactor (TRR). Furthermore, because it is a radiolanthanide; it can be combined with well-known bone-seeking agents such as EDTMP, DOTMP, and zoledronic acid^11^, which have been used to prepare bone-pain-palliatives, to generate new products. However, one drawback is the time-consuming quality control process involved in making pure beta emitter products using liquid scintillation counting with ^143^Pr due to a lack of gamma emission.

There are few reports on the production and application of ^143^Pr. The earliest study was conducted in 1957 by Peppard et al., who reported the efficient isolation of carrier-free ^143^Pr from neutron-bombarded cerium after applying solvent extraction using a system containing 10 M HNO_3_ as an aqueous phase into a 0.75-M or 0.30-M solution of di (2-ethyl hexyl) orthophosphoric acid (HDEHP) in *n*-heptane^12^. In 1963, following Peppard’s report, J. W. Winchester prepared an extractive chromatographic stationary phase using the same extractant of HDEHP held on dichlorodimethyl-silane-treated diatomaceous silica to separate rare earths. The observed separation factors for adjacent rare earths were similar to those of rare earths in a solvent extraction, uniformly close to 2.5^13^.

In other research, Tomitaro Ishimuri et al. separated ^143^Pr from neutron-irradiated uranium by solvent extraction using HEDHP/HNO_3_ system after almost two days of cooling. Then, ^143^Pr was purified after most of the ^143^Ce had decayed^14^. Deptula et al. applied an extraction chromatography (HD_2_EHPA was used as an extractant on resin) following cation exchange chromatography (using resin 5WX4) to separate ^143^Pr from cerium dioxide irradiated in the EWA reactor^15^.

In 1976, Masumitsu Kubota utilized a cation exchange separation method using Diaion SK-1, 100–200 mesh. The chemical yield of the ^143^Pr was over 99%, and its radiochemical purity exceeded 99.99%^16^. The latest reports on the preparation of ^143^Pr were provided by K.V. Vimalnath, who also used an indirect production method by irradiating cerium. The radiochemical separation of ^143^Pr was performed by precipitation; the separated n.c.a-grade ^143^Pr had high radionuclidic purity (> 99.9%) and did not contain any detectable impurities^17–19^.

As mentioned earlier there are not many reports on separation of ^143^Pr. However, solvent extraction, chromatography and precipitation are the most widely used approaches, which are pointed above. Regarding the ease of automation, absence of any organic liquid, and also considering the final chemical/physical-form of product containing ^143^Pr, authors chose the chromatographic approach.

In this study, natural cerium target material is irradiated in Tehran Research Reactor. The calculated optimized time windows for irradiation and cooling the irradiated target material are applied. The well-established extraction chromatography is then applied to isolate ^143^Pr from cerium target material. The optimized parameters of extraction chromatography for the separation of praseodymium from the adjacent lanthanide, cerium, are investigated and determined. The well-known Ln-resin as efficient stationary phase and HNO_3_ as eluent are used^20,21^. Oxidizing properties of cerium were exploited to differentiate the chemical behavior of cerium and praseodymium^22^. Consequently, an efficient and convenient separation of no-carrier-added ^143^Pr from irradiated cerium target material is achieved. Cerium can also be recovered in + 3 oxidation state using ascorbic acid, to be re-used if the costly enriched target material of cerium is used.

## Experimental procedure

### Materials and instruments

Irradiation was performed at Tehran Research Reactor (TRR). The TRR reactor is a 5 MWt open pool design that uses MTR fuel configurations. It is capable of producing a maximum local thermal neutron flux of approximately 1.0 × 10^14^ n/cm^2^ sec within an irradiation box located at the reactor core's center. This irradiation box is used for various activities such as producing radioisotopes, conducting neutron activation analysis, training personnel, and irradiating samples for research purposes. The reactor core itself rests on a grid plate composed of a 9 × 6 array that forms rectangular locations for fuel elements.

Natural cerium (III) nitrate hexahydrate (99.999% trace metals basis) was purchased from Sigma-Aldrich. The extraction resin Ln (100–150 μm) was prepared from Eichrom. All other chemical reagents were in an analytical grade and they were also purchased from Merck. The quartz ampoules, glass chromatography columns, and other disposal fittings were obtained from the NSTRI’s equipment supply workshop. An ultra-low-level liquid scintillation counter (Quantulus 1220) was used for beta counting measurements, the necessary calibrations were performed using ^14^C standard source as an external standard. All aliquots of the ^143^Pr and Ce-isotope samples were mixed with 15 mL of HiSafe3, provided by Perkin-Elmer.

Gamma-ray spectroscopy was performed by a p-type coaxial high-purity germanium detector (HPGe, EGPC 80-200R) coupled with a multichannel analyzer and Gamma 2000 software (Silena). Energy and efficiency calibrations were determined by γ-ray sources traceable to the National Institute of Standards and Technology. A high-performance multichannel laboratory peristaltic pump (DYNAMAX, RP-1) was coupled with an Ln-resin-containing chromatography column and was used to apply suction to the column bed and adjust the output solution fractions volume and flow rate. MATLAB software was utilized to calculate radioactivity.

### Radioactivity calculations for obtaining praseodymium-143 from the neutron irradiation of cerium target material

**Figure 1:**
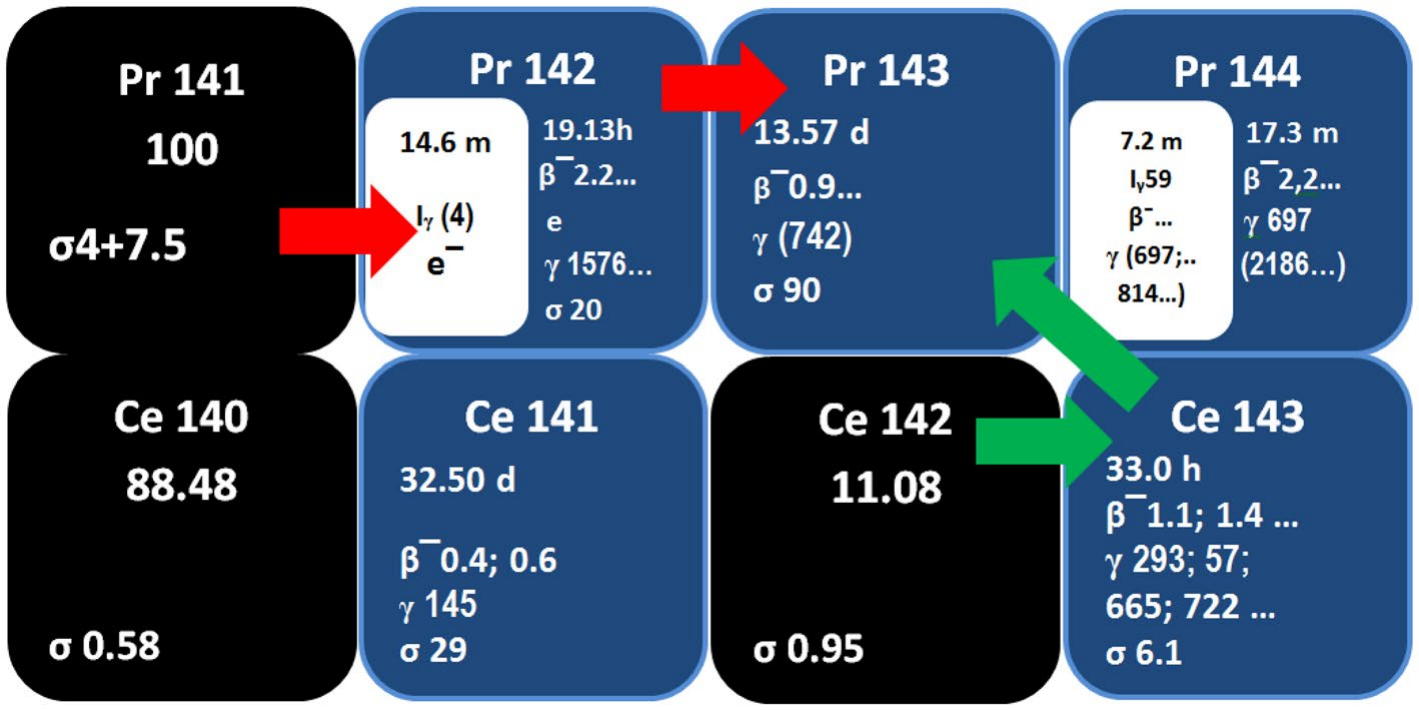
The production of ^143^Pr applying the direct method using Pr-target material (red) and the indirect method using Ce-target material (green).

There are two routes for the production of ^143^Pr through (n,γ) reaction, Fig. 1; the direct method uses Pr-target material (red) and the indirect method uses Ce-target material. This study aims to produce non-carrier-added^143^Pr through the indirect method. The number of nuclei of cerium-143 and praseodymium-143 isotopes created by irradiating 1 mg of natural cerium for 24 h with a neutron flux of 5 × 10^13^ n.cm^−2^ s^−1^ and cooled for 72 h, was calculated using MATLAB software. For calculations, relevant data (cross-section and half-life) from The IAEA Nuclear Data Services were used^23^.

The time-dependent change in nuclear densities arising from neutron activation and transmutation can be expressed using the following system of differential equations:

1 $$\frac{d{N}_{i}(r,t)}{dt}= \varphi \left(r,t\right)\sum_{j}{N}_{j}\left(r,t\right){\sigma }_{j\to i}\left(r\right)+\sum_{k}{N}_{k}\left(r,t\right){\lambda }_{k\to i}\left(r\right)- \varphi \left(r,t\right){N}_{i}\left(r,t\right)\sum_{l}{\sigma }_{i\to l}\left(r\right)- {N}_{i}(r,t)\sum_{n}{\lambda }_{i\to n}$$

Equation (1) constitutes a system of homogeneous first-order ordinary differential equations that describe the time evolution of nuclear densities in the irradiated cerium target.

### Preparation and irradiation of the target material

A solution containing 1 mg of natural cerium nitrate target material was heated in a quartz ampoule under a nitrogen environment to dryness. The open end of each quartz tube was welded and fully closed once the solution had dried. The quartz capsules were then deposited in an aluminum cylinder chamber. Samples were irradiated in the reactor for 24 h with a flux of 5 × 10^13^ cm^−2^ s^−1^. After irradiation, the sample was cooled for 72 h. A 10% nitric acid solution was then added to dissolve the dry precipitate, and the fluid in the quartz capsule was drawn with a syringe and emptied into a vacuum vial. The appropriate quantities of this solution were recorded for gamma and beta spectrum analysis, depending on their activity. All sample and dilution stages were carried out in a glove box in compliance with radiation safety standards.

### Sample analysis

The intended product, ^143^Pr, is a pure-beta emitter and the impurities emit gamma rays; therefore, the purity of the samples was determined using gamma spectroscopy and beta analysis (with liquid scintillation counting method). The quantification of beta radioactivity via liquid scintillation counting is a method employed to gauge the level of beta-emitting nuclides within a sample. This process involves utilizing a fluid scintillation medium (cocktail), which is an aqueous solution comprising a scintillating agent capable of emitting light upon intersection with ionizing radiation. When beta particles are discharged from the isotopes inside the sample and collide with molecules of the scintillating agent suspended in the medium, a flash of light is produced. This light yield is then detected and analyzed to determine the radioactive disintegrations occurring per unit time, thereby allowing calculation of the concentration of beta-emitting isotopes in the original sample. In this work, Wallac Quantulus 1220 counter and OptiPhase HiSafe 3 cocktail were used for beta analysis. Each sample was measured three times to minimize the error.

### Isolation of praseodymium-143 from the irradiated target material

Extraction chromatography with an Ln-resin and an initial mass of target material containing 1 mg natural cerium was employed to separate ^143^Pr from the target material of cerium. This approach is a separation strategy that combines the selectivity of an extracting agent with the speed and convenience of a chromatographic column.

A 22-cm-long column with a diameter of 1 cm containing Ln-resin (150–100 μm) was utilized. The resin slurry in nitric acid entered the column uniformly after the glass wool was placed at the bottom of the pipe. Next, the resin was rinsed with 500 mL of distilled water. The sample comprising cerium and praseodymium was loaded on the column at a flow rate of 8 mL/min after a NaBrO_3_ oxidizing agent had been added. The washing solution, which included 0.01 M nitric acid and 0.05 M oxidizing agent, was then run through the column. This was done to guarantee a higher oxidation state of the cerium of (IV) quantitatively and to perform a column washing step. The radio-lanthanides were eluted from the column by successive changing of the nitric acid through the column as the mobile phase. The eluted fractions were collected from the column in 5-mL quantities.

## Results and discussion

### The calculated and measured produced activities of Ce-143 and its daughter Pr-143

**Figure 2:**
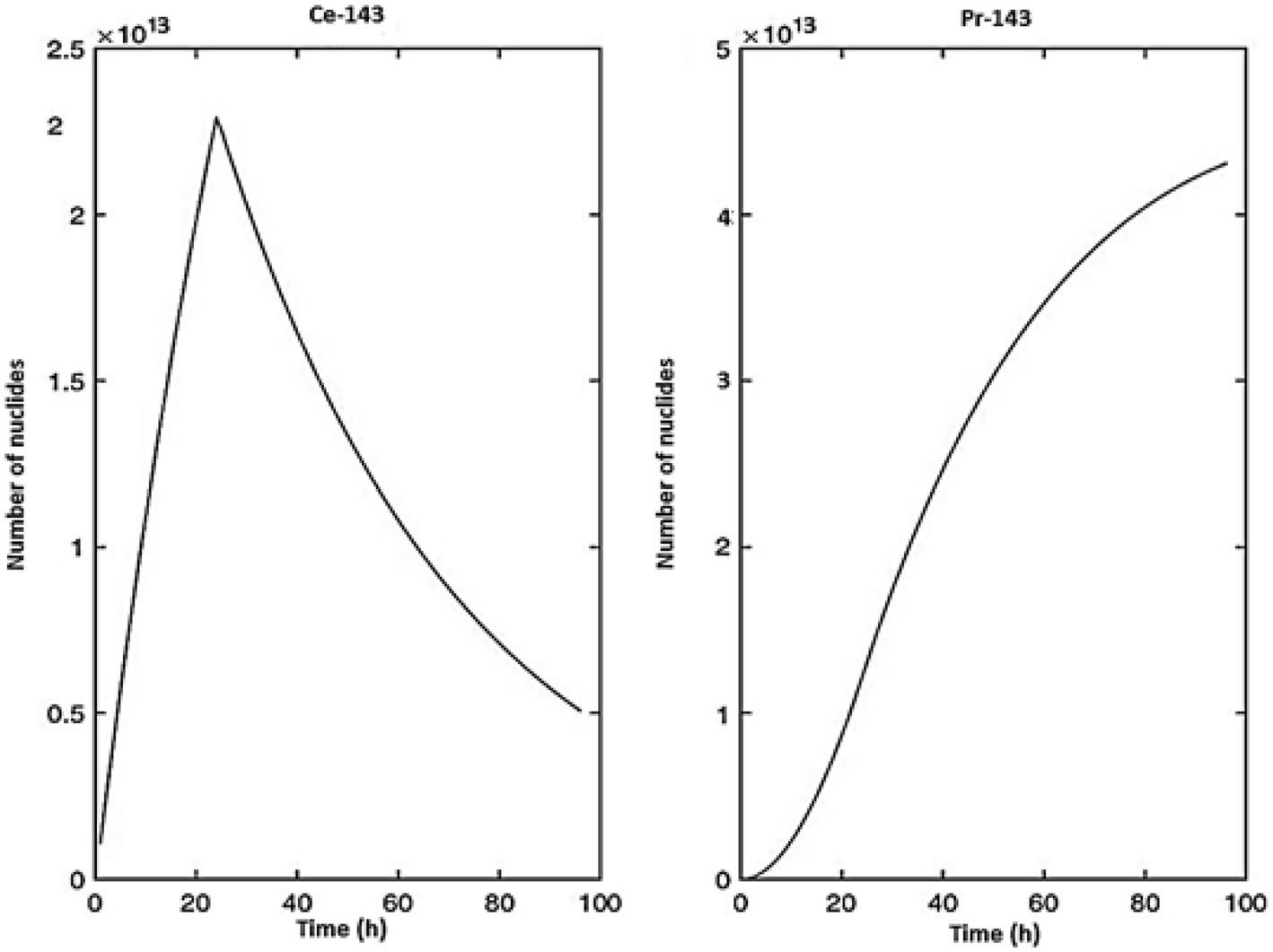
The number of nuclei of ^143^Ce and ^143^Pr isotopes created by irradiating 1 mg of natural cerium for 24 h with a neutron flux of 5 × 10^13^ cm^−2^ s^−1^and cooled for 72 h.

Result of calculations to determine the number of nuclei of cerium-143 and praseodymium-143 isotopes (activities) created by irradiating 1 mg of natural cerium for 24 h with a neutron flux of 5 $\times$ 10^13^ cm^−2^ s^−1^ and cooled for 72 h is depicted in Fig. 2. The 72 h cooling time is necessary for Ce-143 to reach equilibrium with Pr-143 and produce the highest possible amount of Pr-143 from the decay of Ce-143.

The estimated and produced activities of cerium-143 and praseodymium-14 for irradiation and cooling under the specified circumstances are provided in Table 2. Given that the discrepancy between the computational and practical findings is less than 15%, the results appear to be in excellent agreement, and the difference can be attributed to measurement uncertainty, analytical sampling error, and the uncertainty of the time and sample location inside the reactor, which is common.

**Table 2:** The calculated and produced activity for cerium-143 and praseodymium-143.

Isotopes	Calculated activity (MBq)	Measured activity (MBq)	Variance (%)
^143^Ce	29.17	26.34	9.7%
^143^Pr	24.70	21.66	12.3%

### The separation of ^143^Pr from the irradiated cerium-containing material

After 72 h, the optimized separation method was utilized for a 1-mg cerium-containing target material that had been irradiated for 24 h.

Table 2 shows the separation values, Rs, for the cerium/praseodymium pair related to elution from a column with a diameter of 1 cm and different heights of Ln-resin. The inhibition/retention factor of praseodymium (III) and cerium (III) on Ln-resin is very close to each other in different concentrations of nitric acid. Therefore, by increasing the length of the column and as a result the height of the resin has limited enhancing effect on the separation. In this way, doubling and even tripling the length of the column did not lead to a significant increase in lanthanide separation. In order to achieve an efficient separation, cerium was oxidized to (IV) oxidation state. Cerium is the only lanthanide that is stable in (IV) oxidation state in aqueous solution. For the rest of lanthanides, this oxidation state mainly exists in solid samples^24^. In this work, cerium was separated from praseodymium through oxidation method with NaBrO_3_ reagent that will result in better separation of cerium and praseodymium couple.

The distribution coefficient (Kd) values were determined as the ratio of cerium concentrations in the adsorbent and mobile phases. In order to obtain the Kd values, cerium solutions at a fixed concentration of 500 ppb in 0.15 M HNO_3_ was used. Various concentrations of NaBrO_3_, ranging from 0.001 to 0.1 M were added to each sample. Cerium was separated on 50 mg of Ln-Resin for 30 min. The concentration of cerium in the filtrate and eluted solutions were determined by ICP-MS. Table 3 shows the Kd values of cerium in the vicinity of the adsorbent (Ln-resin) as a function of NaBrO3 concentration. It can be seen that with increasing concentration of NaBrO3, Kd values related to Ce increase until the concentration reaches 0.05 M of NaBrO3, at higher concentrations, Kd slightly decreases. The increase in retention/inhibition of Ce on the adsorbent, with the increase of NaBrO3 concentration up to 0.05 M, is most likely due to the oxidation of cerium (III) to cerium (IV). Since HDEHP is a cationic extractant, at concentrations higher than 0.05 M NaBrO3 it decreases the retention/inhibition of cerium from Ce on the resin, which is probably the result of the presence of sodium cations that interact with cerium to sit on the extraction sites (Table 4).

**Table 3:** Resolution values, Rs, for the cerium/praseodymium pair for elution from a column with a diameter of 1 cm and different lengths of Ln-resin height.

Parameter	Height of Ln-resin in column		
	12 cm	22 cm	39 cm
Separation values (Rs)	0.1	0.2	0.2

**Table 4:** Cerium K_d_ values as a function of HNO_3_ concentration.

In the presence of variable NaBrO_3_ HNO_3_ constant concentration of 0.15 M	K_d_	In the absence of NaBrO_3_ variable HNO_3_	K_d_
0.001	~ 20	0.01	~ 20,000
0.01	50	0.04	~ 2000
0.05	200	0.1	~ 200
0.1	35	0.5	~ 10

Subsequent column studies showed that the oxidation of cerium (III) to cerium (IV) can be performed directly on the column and the reduction of cerium (IV) to cerium (III) can be performed on the column using 0.05 M ascorbic acid. The significant increase in Kd values as a result of the addition of NaBrO3 shows a significant potential for improving Ce/Pr separation factors using this approach.

**Figure 3:**
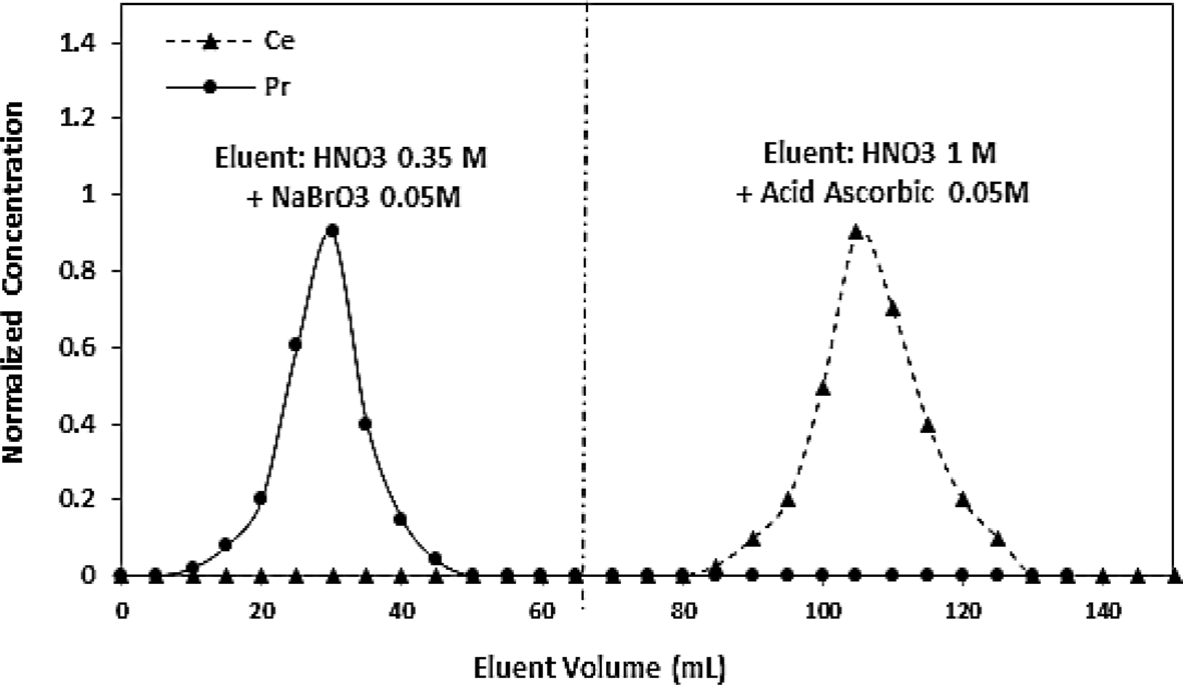
Pr/Ce separation in optimal conditions, a column with a diameter of 1 cm and containing resin with a height of 22 cm, resin with dimensions of 100 to 200 μm.

Figure 3 shows the separation of praseodymium and cerium under optimal conditions (a column with a diameter of 1 cm and containing resin with a height of 22 cm, resin with dimensions of 100 –200 μm). Separation of cerium base from praseodymium was easily achieved using NaBrO_3_/ascorbic acid system.

The eluted samples from the column were submitted for nuclear analysis. The results of this analysis are shown in the next section.

### Quality control: radiochemical and radionuclidic purity

The product, ^143^Pr, does not emit any gamma-rays, while the impurities, including ^141^Ce and ^143^Ce, do. Thus, during the quality control studies, all eluted fractions were analyzed using gamma spectroscopy and beta scintillation methods.

**Figure 4:**
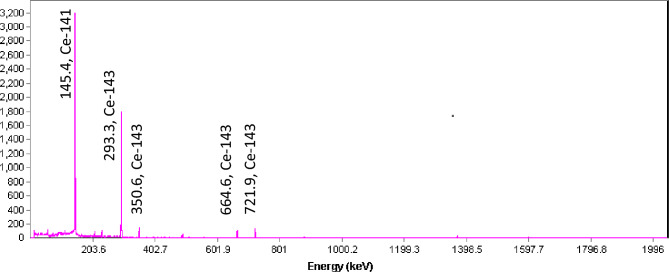
Gamma spectra of the solution containing irradiated cerium target material,

In the first step, gamma spectrometry was used to count all eluted fractions at various stages of separation in equal volumes in containers with similar geometries. Although the presence or absence of ^143^Pr in a sample could not be confirmed, the quantities of the cerium-isotopes of ^141^Ce and ^143^Ce in each sample could be determined, as they have different gamma-ray energies (Fig. 4).

In the second step, a portion of each sample was extracted and analyzed using the liquid scintillation method. The radioactive sample was delivered in a special 20-ml vial with 10 ml double distilled water. Then, 10 ml of cocktail liquid (the scintillator) was added. The liquid scintillation counting (LSC) is the most accurate method for measuring pure-beta-emitter content. It can be used to count any radioisotope, but it is expensive and requires specific counting solutions to be prepared. Because the beta beam, unlike the gamma-ray, has a continuous spectrum, it is impossible to detect separate beta radionuclides in a sample; in other words, this instrument measures the total beta. Since the samples might have included cerium isotopes (emission of beta plus gamma-ray) and ^143^Pr (emission of pure beta with no gamma-ray), the sample counting is not correct with LSC alone, so the sample was counted with both gamma spectrometry and LSC.

Cerium isotopes or ^143^Pr are involved in the measured activity. However, because the presence of cerium in the samples could be determined using gamma spectrometry, the cerium isotopes in the samples were measured first, and then the total beta was determined to measure the ^143^Pr. The gamma counts of each fraction were measured using gamma spectrometry, and the beta counts of each fraction were measured using the liquid scintillation counter (Table 5).

**Table 5:** Activity concentrations of the eluted fractions.

Fraction number	Ce-143 (MBq) γ, 145 keV	Ce-141 (MBq) γ, 293 keV	Gross beta (MBq)
1	1.59 ± 0.11	1.35 ± 0.12	2.14 ± 0.13
2	0.07 ± 0.01	0.08 ± 0.01	14.29 ± 0.69
3	0.22 ± 0.02	0.19 ± 0.02	3.01 ± 0.17
4	0.19 ± 0.02	0.16 ± 0.02	1.63 ± 0.10
5	0.11 ± 0.02	0.09 ± 0.01	0.93 ± 0.05
6	0.68 ± 0.08	0.58 ± 0.06	0.86 ± 0.06
7	0.64 ± 0.08	0.58 ± 0.06	0.82 ± 0.06
8	0.86 ± 0.07	0.71 ± 0.08	1.45 ± 0.10
9	21.59 ± 1.13	19.29 ± 1.31	27.50 ± 1.23
10	0.39 ± 0.05	0.32 ± 0.04	2.24 ± 0.12

**Figure 5:**
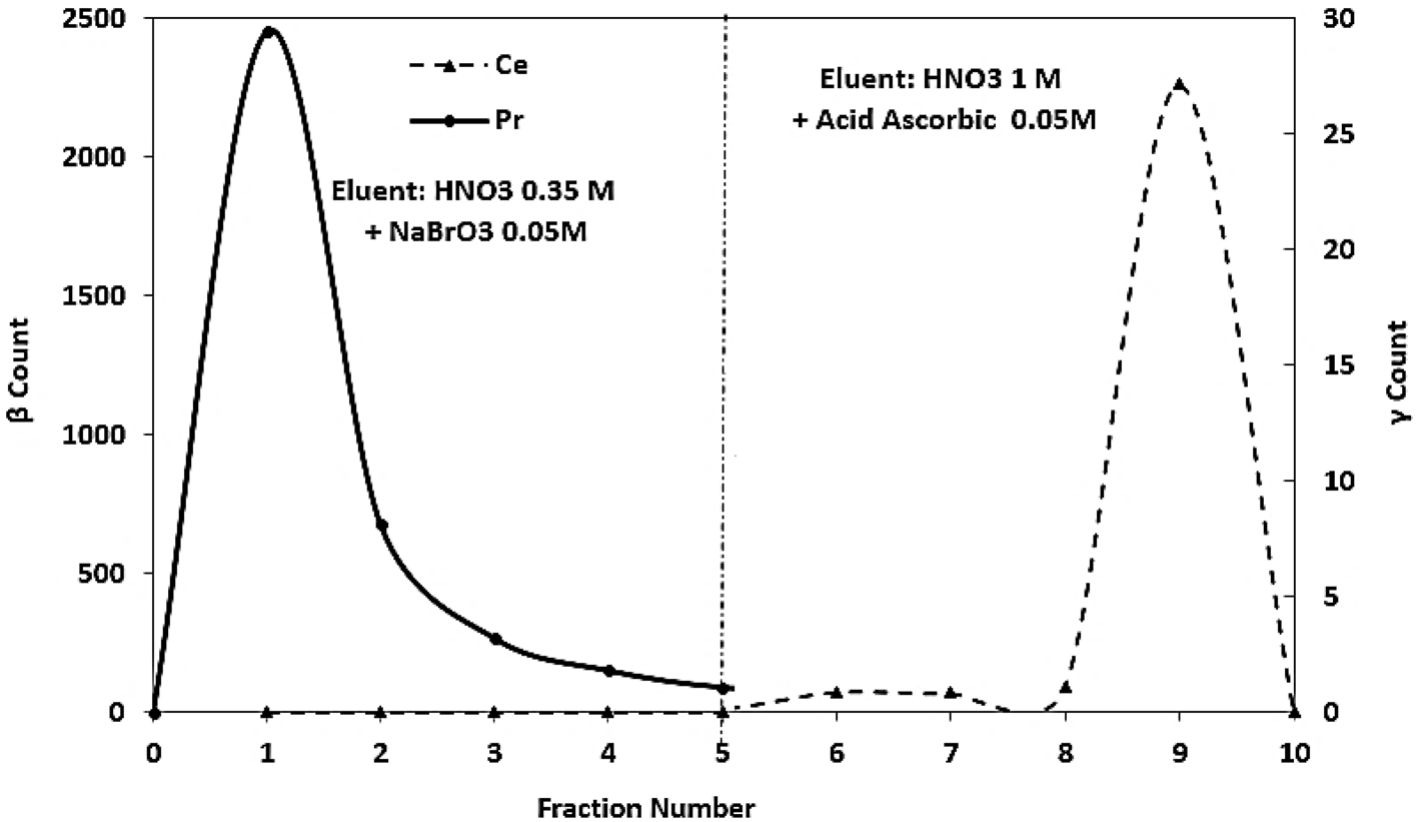
The chromatogram obtained from non-carrier-added ^143^Pr separation from the irradiated target material of natural cerium.

Figure 5 also shows the resultant chromatogram of non-carrier-added ^143^Pr separation from the irradiated target material of natural cerium.

Results obtained from separation of n.c.a.^143^Pr from the irradiated target material of natural cerium show the radionuclidic purity of the isolated ^143^Pr which are greater than 99%.

Thin layer chromatography was applied using Whatman paper and a 0.1 M DTPA solution to assess radiochemical purity. One beta count peak appeared at R_f_ = 0.9. It was concluded that the product contained only one radionuclidic species, namely ^143^Pr salt (nitrate), with a purity of above 99%. A sample of ^143^Pr was passed through a 2-ml cartridge containing Ln-resin, and ^143^Pr was then eluted with 0.35 M nitric acid to determine its chemical purity.

After cooling, an elemental analysis was conducted to analyze the ^143^Pr-nitrate salt sample after five to seven half-lives, or about three months (CHN and ICP). ICP/CHN, or Inductively Coupled Plasma/Carbon Hydrogen Nitrogen analysis, is an analytical technique used to determine the concentration of elements in a sample. The sample is first injected into the instrument. The sample solutions are then nebulized with argon gas and injected into a plasma torch, where the atoms and ions are excited to emit electromagnetic radiation at wavelengths characteristic of specific elements. The intensities of these emitted wavelengths are measured and can be correlated to the original concentration of elements in the samples. In this method, no cerium was observed in the sample with the sensitivity of ICP by analyzing the solution. The chemical purity was also over 99%. The primary sources of chemical contamination in the isolation of radionuclides during the chromatographic process were polymer resins, their detached fragments, and the extracting agents attached to them. Fortunately, these contaminants were not observed in the final product of our research.

## Conclusion

In this study, we developed and optimized a process for producing high purity non-carrier-added 143Pr via neutron activation of natural cerium oxide and subsequent chromatographic purification. Calculations assisted in determining the irradiation and cooling parameters needed to indirectly produce 143Pr through decay of 143Ce. Extraction chromatography using Ln-resin efficiently isolated 143Pr from the irradiated target material with radionuclidic purity exceeding 99%.

Additional quality control analyses confirmed the radiochemical and chemical purity of the purified 143Pr nitrate product met regulatory standards for potential medical applications. With its half-life of 13.6 days and beta energy of 0.937 MeV, ^143^Pr shows promising properties for palliative bone pain therapy.

While further preclinical investigations are still needed, the convenient production method presented here establishes a viable path forward for exploring potential clinical uses of ^143^Pr. Optimizing target material and irradiation parameters could help improve yield. Investigations into complexing ^143^Pr with bone-seeking agents could yield radiopharmaceuticals with optimized pharmacokinetics.

This work demonstrates the feasibility of utilizing ^143^Pr as a pain palliative through an optimized production and purification process. Continued research has the potential to translate this radioisotope into clinically valuable targeted radiotherapies for the palliation of bone metastasis pain.

Scaling up the production method described in the study to provide the required amounts of n.c.a. ^143^Pr for nuclear medical applications would involve optimizing the irradiation parameters, such as target material mass and irradiation time, to increase the yield. The purification process, including extraction chromatography and subsequent purification steps, would need to be adapted for larger volumes, while maintaining high purity. Quality control analysis should be conducted to ensure the chemical and radiochemical purity of the scaled-up production. altogether, with careful adjustments and optimizations, it is feasible to scale up the production of high-purity ^143^Pr to meet the demands of nuclear medical applications, while considering regulatory and practical considerations.

## Data Availability

My manuscript has no associated data or the data will not be deposited. The author confirms that all data generated or analyzed during this study are included in this manuscript.

## References

[1] Ayati N, Aryana K, Jalilian A, Hoseinnejad T, Bahrami Samani A, Ayati Z, Shariati F, Zakavi R (2013). Treatment efficacy of ^153^Sm-EDTMP for painful bone metastasis. Asia. Ocean. J. Nucl. Med. Biol..

[2] Kraeber-Bodéré F, Campion L, Rousseau C, Bourdin S, Chatal JF, Resche I (2000). Treatment of bone metastases of prostate cancer with strontium-89 chloride: efficacy in relation to the degree of bone involvement. Eur. J. Nucl. Med..

[3] Fettich J, Padhy A, Nair N, Morales R, Tanumihardja M, Riccabonna G, Nair G (2003). Comparative clinical efficacy and safety of phosphorus-32 and strontium-89 in the palliative treatment of metastatic bone pain: Results of an IAEA Coordinated Research Project. World J. Nucl. Med..

[4] Solá GAR, Argüelles MG, Bottazzini DL, Furnari JC, Parada IG, Rojo A, Ruiz HV (2009). Lutetium-177-EDTMP for bone pain palliation: Preparation, biodistribution and pre-clinical studies. Radiochim. Acta..

[5] Nienke JM, Klaassen Mark J, Arntz G, Arranja A, Roosen Joey W, Nijsen JF (2019). The various therapeutic applications of the medical isotope holmium-166: A narrative review. EJNMMI. Radiopharm. Chem..

[6] Döbert N, Martin H, Kranert WT, Menzel C, Klein SA, Mose S, Grünwald F (2003). Re-186 HEDP conditioning therapy in patients with advanced acute lymphoblastic leukemia before allogeneic bone marrow transplantation. Clin. Nucl. Med..

[7] Manafi-Farid R, Masoumi F, Divband Gh, Saidi B, Ataeinia B, Hertel F, Schweighofer-Zwink G, Morgenroth A, Beheshti M (2020). Targeted palliative radionuclide therapy for metastatic bone pain. J. Clin. Med..

[8] Alavi M, Omidvari S, Mehdizadeh A, Jalilian AR, Bahrami-Samani A (2015). Metastatic bone pain palliation using ^177^Lu-ethylenediaminetetramethylene phosphonic acid. World. J. Nucl. Med..

[9] Chakraborty S, Balogh L, Das T, Polyak A, Andocs G, Mathe D (2016). Evaluation of ^177^Lu-EDTMP in dogs with spontaneous tumor involving bone: Pharmacokinetics, dosimetry and therapeutic efficacy. Curr. Radiopharm..

[10] Sharma S, Singh B, Koul A, Rai Mittal B (2017). Comparative therapeutic efficacy of ^153^Sm-EDTMP and ^177^Lu-EDTMP for bone pain palliation in patients with skeletal metastases: Patients’ pain score analysis and personalized dosimetry. Front. Med (Lausanne).

[11] Kuźnika A, Październiok-Holewaa A, Jewula P, Kuźnik N (2020). Bisphosphonates—Much more than only drugs for bone diseases. Eur. J. Pharmacol..

[12] Peppard DF, Mason GW, Moline SW (1957). The use of dioctyl phosphoric acid extraction in the isolation of carrier-free ^90^Y, ^140^La, ^144^Ce, ^143^Pr, and ^144^Pr. J. Inorg. Nucl..

[13] W. Winchester J, (1963). Rare earth chromatography using bis-(2-ethylhexyl) orthophosphoric acid. J. Chromatogr. A..

[14] Ishimori T, Kobayashi Y (1965). Praseodymium-143 from neutron-irradiated uranium. J. Nucl. Sci. Technol..

[15] Deptula C, Makowski H, Wiza J (1971). Preparation of ^141^Ce and ^143^Pr ferom cerium dioxide irradiated in the new reactor. Nukleonika.

[16] Kubota M (1976). Preparation of high purity praseodymium-143 from neutron irradiated cerium oxide by cation-exchange separation. J. Nucl. Sci. Technol..

[17] Vimalnath KV, Das MK, Venkatesh M, Ramamoorthy N (2005). Prospects and problems in the production of ^143^Pr for radionuclide therapy applications. Radiochim. Acta.

[18] Vimalnath, K.V, Das, M.K., Venkatesh, M. & Ramamoorthy, N. Production logistics and prospects of ^142^Pr and ^143^Pr for radionuclide therapy (RNT) applications. In *5th International Conference on Isotopes, by MEDIMOND S.r.l. F425R0149*. 103–108 (2005).

[19] Vimalnath, K.V., Viju, C., Sharad, P.L., Rajeswari, A., Thakare, S.V., Shivarudrappa, V., Joshi, P.V., Venkatesh, M. *Reactor Production of n.c.a Grade *^*143*^*Pr, *^*161*^*Tb and *^*111*^*Ag Radionuclides for Radionuclide Therapy Applications Web.Conference, INIS Volume 41, INIS Issue 06, Indian Nuclear Society*. Vol. 384. 583–584. *International Conference on Peaceful Uses of Atomic Energy New Delhi* (*India*), 29 Sep–1 Oct 2009 (2009).

[20] McAlister, D. R. & Horwitz, E. P. *Characterization of Extraction of Chromatographic Materials Containing Bis(2‐Ethyl‐1‐Hexyl) Phosphoric Acid, 2‐Ethyl‐1‐Hexyl (2‐Ethyl‐1‐Hexyl) Phosphonic Acid, and Bis(2,4,4‐Trimethyl‐1‐Pentyl) Phosphinic Acid*. 757–769 (2007).

[21] Arrigo L, Jiang J, Finch Z, Bowen J, Beck C, Friese J, Greenwood L, Seiner B (2021). Development of a separation method for rare earth elements using LN resin. J. Radioanal. Nucl. Chem..

[22] (Russ) Knapp, F. F. & Dash, A. *Radiopharmaceuticals for Therapy*. 87 (2016).

[23] IAEA Nuclear Data Services, International Atomic Energy Agency. https://www-nds.iaea.org/ (2024).

[24] Greenwood NN, Earnshaw A (1997). Chemistry of the Elements.

